# Trend in case detection rate for all tuberculosis cases notified in Ebonyi, Southeastern Nigeria during 1999-2009

**DOI:** 10.11604/pamj.2013.16.11.680

**Published:** 2013-09-12

**Authors:** Kingsley Nnanna Ukwaja, Isaac Alobu, Ngozi Appolonia Ifebunandu, Chijioke Osakwe, Chika Igwenyi

**Affiliations:** 1 Department of Internal Medicine, Ebonyi State University Teaching Hospital Abakaliki, Ebonyi State, Nigeria; 2 National Tuberculosis and Leprosy Control Programme, Ministry of Health, Ebonyi State Nigeria; 3 Department of Medicine, Federal Medical Centre, Abakaliki, Nigeria; 4 World Health Organisation, Southeast zone, Nigeria

**Keywords:** Tuberculosis, TB control, DOTS, case detection, Nigeria

## Abstract

Unlike previous annual WHO tuberculosis reports that reported case detection rate for only smear-positive tuberculosis cases, the 2010 report presented case detection rate for all tuberculosis cases notified in line with the current Stop TB strategy. To help us understand how tuberculosis control programmes performed in terms of detecting tuberculosis, there is need to document the trend in case detection rate for all tuberculosis cases notified in high burden countries. This evidence is currently lacking from Nigeria. Therefore, this study aimed to assess the trend in case detection rate for all tuberculosis cases notified from Ebonyi state compared to Nigeria national figures. Reports of tuberculosis cases notified between 1999 and 2009 were reviewed from the Ebonyi State Ministry of Health tuberculosis quarterly reports. Tuberculosis case detection rates were computed according to WHO guidelines. 22, 508 patients with all forms of tuberculosis were notified during the study. Case detection rate for all tuberculosis rose from 27% in 1999 to gradually reach a peak of 40% during 2007 to 2008 before a slight decline in 2009 to 38%. However, the national case detection rate for all tuberculosis cases in Nigeria rose from 7% in 1999 and progressively increased to reach a peak of 19% during 2008 and 2009. Since the introduction of DOTS in Ebonyi, the programme has achieved 40% case detection rate for all tuberculosis cases - about 20% better than national figures. However, with the current low case detection rates, alternative mechanisms are needed to achieve the current global stop- TB targets in Nigeria.

## Introduction

In 2009, there were an estimated 9.4 million incident cases of tuberculosis (TB) worldwide [1]. Nigeria ranks fourth among the 22 high burden tuberculosis countries [1]. The WHO estimates that, the incidence of all forms TB in Nigeria stands at 311/100,000 population in 2009 [2]. The National Tuberculosis and Leprosy Control programme (NTBLCP) was started in 1991 and the Directly Observed Treatment Short Course (DOTS) strategy adopted in 1993 [2]. The DOTS strategy was gradually scaled up nationwide and was introduced to the tuberculosis control programme in Ebonyi state since its creation in 1996. Key strategies of the NTBLCP are early case detection and treatment in order to interrupt transmission, reduce morbidity / mortality and prevent the emergence of drug resistance amongst TB patients [2].

One of the main indicators to assess the quality of a tuberculosis control programme is case detection rate (CDR) [1]. Increased case detection will decrease transmission rapidly provided cure rates are high. Unlike previous annual WHO TB reports that reported case detection rate for only smear-positive TB cases, the 2010 report presented case detection rate for all TB cases notified by various countries [1]. The considerable attention previously given to smear-positive CDR was in line with the two principal global targets. The targets of reaching a CDR of ≥ 70% and a treatment success rate of ≥ 85% among sputum smear-positive cases of pulmonary TB by 2000. These were set by the Forty-fourth World Health Assembly in 1991, with the target year subsequently reset to 2005 [1]. Major attention was given to detecting and curing people with sputum smear-positive pulmonary TB because they are most infectious - and thus the most likely without proper treatment to cause further transmission of TB [1].

There are several reasons for the current change in TB reportage; National TB Programs in all countries are diagnosing, notifying and treating people with all forms of TB, not just those with sputum smear-positive TB [1]. Also, the Stop TB strategy launched in 2006; emphasizes the detection and treatment of people with all forms of TB [3]. Similarly, the 2015 global targets set within the context of the millennium development goals (MDGs) and by the Stop-TB Partnership, which are now the focus of national and international efforts to control TB, are defined in terms of reductions in the disease burden caused by all forms of TB [4].

To help us understand how TB control programmes performed in terms of detecting TB and since regional differences in the proportion of TB cases detected may vary, there is need to document the trend in CDR for all TB cases notified in high TB burden countries. This evidence is currently lacking from Nigeria. Therefore, this study is aimed to assess the CDR for all TB cases notified from Ebonyi state compared to national figures during 1999 to 2009.

## Methods

**Study setting:** Ebonyi state is one of the 36 states of Nigeria located in the southeast geopolitical zone with an estimated population of over 2.5 million people [5]. The state has 13 local government areas (LGA) with 130 health care facilities currently providing DOTS services. All DOTS units have standard unit registers from the NTBLCP. Each LGA have a Tuberculosis and Leprosy control supervisor responsible for managing and coordinating TB and leprosy control activities in the local government as well as keeping up-to-date and accurate record of activities there. They also provide monthly report to the State Tuberculosis and Leprosy control officer whose responsibilities among others include collection, collation and analysis of data on leprosy and TB activities in the state and dissemination of reports.

**Design and data collection:** For the purpose of this study, we undertook a retrospective review of the CDR for all TB cases notified by all the local government areas in the state during 1999 - 2009. The districts quarterly reports of all TB cases notified during the period were reviewed.

**Statistical analysis:** The collected data were recorded and analysed using Microsoft Excel software. The case detection rate (CDR) was calculated as previously documented [1, 6] by dividing the notification rate (per 100,000) of all cases per year for Ebonyi state and Nigeria by the WHO ? estimated number of all (incident) cases per 100000 population in the same year for Nigeria.

**Ethical Approval:** Ethical approval was not required as this is a retrospective study and this report is part of standard public health practice. The National Tuberculosis and Leprosy Control Programme Office, Ebonyi State, Nigeria, granted permission to publish this report.

## Results

**Figure 1:**
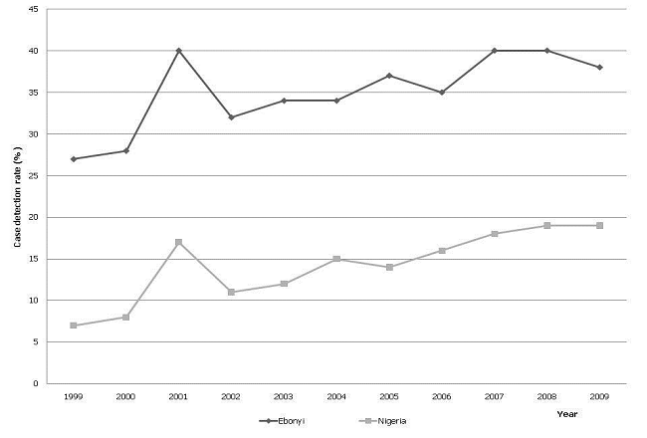
Case detection rate for all tuberculosis cases notified during 1999 – 2009 in Ebonyi and Nigeria

Over the 11-year period from 1999 to 2009, 22, 508 patients with all forms of TB were notified. Trend in CDR for all TB cases notified in Ebonyi state and Nigeria are as shown in Figure 1. Case detection rate for all TB rose from 27% in 1999 to 40% in 2001 and then declined to 32% in 2002. It then rose gradually from 2002 to reach a peak of 40% during 2007 to 2008 before a slight decline in 2009 to 38% (Figure 1). However, the national case detection rate for all TB cases in Nigeria rose from 7% in 1999 to 17% in 2001 and then declined to 11% in 2002. It then progressively increased from 2002 to reach a peak of 19% during 2008 and 2009 - thus national CDR remained persistently below 20% (Figure 1). The increase in CDR in Ebonyi in 2009 compared to 1999 levels, correlated with the decentralisation and expansion of the DOTS services to more health facilities in the state, which increased from 21 in 1999 to 102 in 2009 (R^2^ = 0.42).

## Discussion

The results of our study show that there has been an increase in CDR for all TB cases in parallel to the expansion and decentralisation of DOTS to lower treatment units in Ebonyi state. The case detection rate for all forms of TB showed a significant increase in the first eight years during the study mainly due to the expansion of DOTS services, and then it stabilized after reaching a peak of 40%. The most likely explanation for the initial (2001) increase in the cases detected in the state is due to improved diagnostic set-up and decentralisation of the diagnostic services, which resulted in registration of a large backlog of cases in the early periods [6, 7].

Also, the observed trend in CDR might also be partly explained by an increase in the incidence of active TB fuelled by the HIV epidemic and improved case finding of TB among HIV patients and vice versa. However, the CDR seems to have leveled off in 2007–2009, despite a remarkable increase in the number of DOTS centres. During these years, an increase in case detection due to improved case finding might have been offset by an actual decrease in the incidence of active TB due to improved case holding and reduced transmission. The trend compares favorably with earlier reports in Ethiopia [6, 7].

The most likely reason while the TB control program in Ebonyi performed better than Nigeria is because DOTS services were introduced in southern Nigeria a decade before it was introduced in the north [8]. Thus, regional differences in case notification for all TB cases might mask progress made in detecting TB in other regions.

Since our study was based on quarterly reports from the NTBLCP office in Ebonyi State, we cannot exclude the possibility of poor recording and reporting systems. However, this has been reduced via several training and re-training sessions on TB recording and reporting using standardized forms / registers organized by the NTBLCP for staff working in all DOTS centres in the state.

## Conclusion

In conclusion, the introduction and expansion of DOTS in Ebonyi State has led to a significant increase in CDR - 20% better than national figures. There is need to increase the CDR to achieve the current global target of detecting all TB cases, and this warrants evaluation of alternative intensified case-finding mechanisms. Lessons learnt from such case finding strategies in other poor resource settings in Africa should be explored in Nigeria.
